# Synthesis and crystal structure of Na_4_Ni_7_(AsO_4_)_6_

**DOI:** 10.1107/S2056989016005417

**Published:** 2016-04-05

**Authors:** Rénald David

**Affiliations:** a Laboratoire de Réactivité et Chimie des Solides, Université de Picardie Jules Verne, CNRS UMR 7314, 33 rue Saint Leu, 80039 Amiens, France

**Keywords:** crystal structure, nickel arsenate, ceramic synthesis

## Abstract

The title compound, tetra­sodium hepta­nickel hexa­arsenate, was obtained by ceramic synthesis and crystallizes in the monoclinic space group *C*2/*m*. The asymmetric unit contains seven Ni atoms of which two have site symmetry 2/*m* and three site symmetry 2, four As atoms of which two have site symmetry *m* and two site symmetry 2, three Na atoms of which two have site symmetry 2, and fifteen O atoms of which four have site symmetry *m*. The structure of Na_4_Ni_7_(AsO_4_)_6_ is made of layers of Ni octa­hedra and As tetra­hedra assembled in sheets parallel to the *bc* plane. These layers are inter­connected by corner-sharing between NiO_6_ octa­hedra and AsO_4_ tetra­hedra. This linkage creates tunnels running along the *c* axis in which the Na atoms are located. This arrangement is similar to the one observed in Na_4_Ni_7_(PO_4_)_6_, but the layers of the two compounds are slightly different because of the disorder of one of the Ni sites in the structure of the title compound.

## Abstract 2

The structure of Na_4_Ni_7_(AsO_4_)_6_ is made of layers of Ni octa­hedra and As tetra­hedra assembled in sheets parallel to the *bc* plane. These layers are inter­connected by corner-sharing between Ni octa­hedra and As tetra­hedra. This linkage creates tunnels running along the *c* axis in which the Na atoms are located.

## Chemical context   

Although the structures of transition metal phosphates have been widely investigated during the last decades, very little work has been done on comparable arsenates due to the toxicity of arsenic. The latter phases can exhibit, however, peculiar properties. BaCo_2_(AsO_4_)_2_ is a good example of a quasi-2D system with a magnetically frustrated honeycomb lattice (Regnault *et al.*, 1977 ▸). BaCoAs_2_O_7_ appears as the first example of a magnetization step promoted by a structural modulation (David *et al.*, 2013*a* ▸). LiCoAsO_4_ shows reversible electrochemical activity at high potential (Satya Kishore & Varadaraju, 2006 ▸). Moreover, a recent study reveals the inter­est of arsenate groups in playing the role of efficient disconnecting units in the magnetic compound BaCo_2_(As_3_O_6_)_2_·H_2_O, being the first pure inorganic compound with slow spin dynamics (David *et al.*, 2013*b* ▸). From the crystal chemistry point of view, substitution of phosphate by arsenate gives the possibility of stabilizing new phases. For example, NaNiPO_4_ crystallizes with the maricite structure (Senthilkumar *et al.*, 2014 ▸), whereas NaNiAsO_4_ has a honeycomb layer structure (Range & Meister, 1984 ▸). In this study, we describe the structure of Na_4_Ni_7_(AsO_4_)_6_ and compare it with its phosphate analogue.

## Structural commentary   

**Figure 1:**
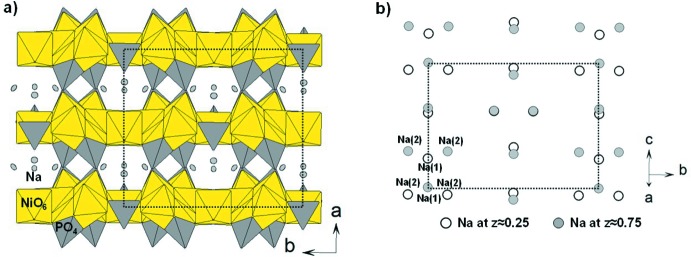
Description of the crystal structure of Na_4_Ni_7_(AsO_4_)_6_ with (*a*) a view of the stacking and (*b*) a view of the Na layers. The dotted lines show the cell edges. Displacement ellipsoids are drawn at the 50% probability level.

**Figure 2:**
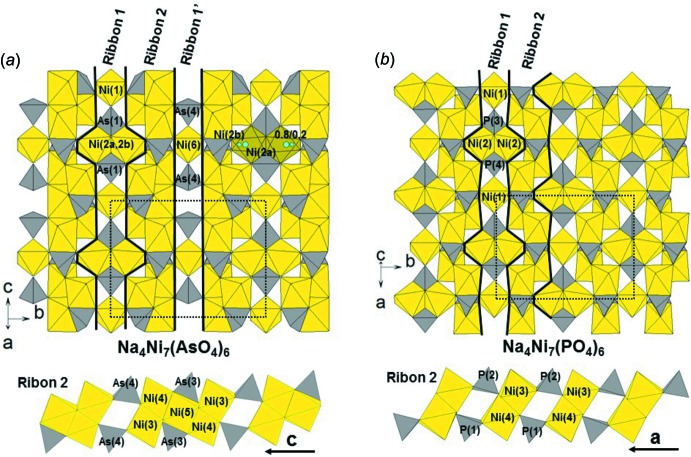
Description of the layers (top) and the ribbon 2 (bottom) of (*a*) Na_4_Ni_7_(AsO_4_)_6_ and (*b*) Na_4_Ni_7_(PO_4_)_6_. The dotted lines show the cell edges.

The structure of the title compound Na_4_Ni_7_(AsO_4_)_6_ is quite similar to the one of the phosphate homologue Na_4_Ni_7_(PO_4_)_6_. Both are made of inter­connected Ni_7_(*X*O_4_)_6_ layers with tunnels in between where the Na atoms are located, as shown in Fig. 1 ▸ *a*. The arrangement of NiO_6_ and *X*O_4_ in the layer is, however, slightly different, as evidenced in Fig. 2 ▸. As described by Moring & Kostiner (1986 ▸), Na_4_Ni_7_(PO_4_)_6_ layers are made of parallel ribbons (called ribbon 1) containing Ni1, Ni2, P3 and P4 polyhedra. These ribbons 1 are inter­connected by another kind of ribbon (called ribbon 2) made of dimers consisting of edge-sharing NiO_6_ octahedra (Ni3 and Ni4). The latter are linked to PO_4_ tetrahedra (P1 and P2) by edge- and corner-sharing. The difference between the two compounds is associated with the possibility of the Ni2 atom in Na_4_Ni_7_(PO_4_)_6_ occupying two octa­hedral sites. The first site, belonging to ribbon 1, is equivalent to the Ni2*a* site in Na_4_Ni_7_(AsO_4_)_6_. The other, equivalent to the Ni2*b* and Ni5 sites in Na_4_Ni_7_(AsO_4_)_6_, belongs to ribbon 2, forming penta­mers of edge-sharing NiO_6_ octa­hedra. The layers of the title compound Na_4_Ni_7_(AsO_4_)_6_ can thus be described with three kinds of ribbons, as shown in Fig. 2 ▸. The linkage between the layers is done by corner-sharing between NiO_6_ and AsO_4_ units of two consecutive ribbons 2 along the stacking axis (Fig. 1 ▸ *a*). This linkage is identical to the one of the phosphorus homologue. However, since in Na_4_Ni_7_(AsO_4_)_6_ layers are made of three different kinds of ribbons, two adjacent layers are shifted to align ribbon 1 with ribbon 1′. That is why in Na_4_Ni_7_(AsO_4_)_6_ the stacking axis is roughly doubled compared to Na_4_Ni_7_(PO_4_)_6_ [*c* = 6.398 (2) Å *versus* *a* = 14.5383 (11) Å in the title structure]. It implies two different kinds of Na layers, as shown in Fig. 1 ▸ *b*.

## Synthesis and crystallization   

Sodium carbonate (>99.5%), arsenic oxide (99%) and nickel sulfate hexa­hydrate (>99.9%) were purchased from Sigma–Aldrich. They were used as received without further purification. Reagents were ground together in stoichiometric ratio in an agate mortar. The obtained mixture was pelletized, placed in an alumina boat and annealed at 573 K for 1h. The obtained mixture was reground, pelletized and heated at 1073 K (5 K min^−1^) for 48 h, after which the alumina boat was removed from the furnace and cooled to room temperature. The brown crystals of the title compound were isolated by hand.

## Refinement details   

Crystal data, data collection and structure refinement details are summarized in Table 1 ▸. The (001) reflection, affected by the beamstop, has been removed from the refinement. Another reflection (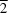
01), flagged as potentially affected by the beamstop, was in fact not and was kept in the refinement. After positioning and refining all the atom positions except Ni2*b*, the difference Fourier map revealed residual density (≃8 e Å^−3^) near Ni2*a* (at ≃0.6 Å). It was refined introducing a second position Ni2*b* with complementary occupation. The occupancy ratio was refined to 0.80 (4):0.20 (4) for the Ni2*a*/Ni2*b* site, constraining the sum to be equal to 1.

## Supplementary Material

Crystal structure: contains datablock(s) global, I. DOI: 10.1107/S2056989016005417/vn2109sup1.cif

Structure factors: contains datablock(s) I. DOI: 10.1107/S2056989016005417/vn2109Isup2.hkl

CCDC reference: 1471603

Additional supporting information:  crystallographic information; 3D view; checkCIF report

## Figures and Tables

**Table 1:** Experimental details

Crystal data	
Chemical formula	Na_4_Ni_7_(AsO_4_)_6_
*M*_r_	1336.3
Crystal system, space group	Monoclinic, *C*2/*m*
Temperature (K)	293
*a*, *b*, *c* (Å)	14.5383 (11), 14.5047 (11), 10.6120 (8)
β (°)	118.299 (2)
*V* (Å^3^)	1970.3 (3)
*Z*	4
Radiation type	Mo *K*α
μ (mm^−1^)	16.76
Crystal size (mm)	0.07 × 0.06 × 0.04

Data collection	
Diffractometer	Bruker D8 Venture
Absorption correction	Multi-scan (*SADABS*; Bruker, 2015 ▸)
*T*_min_, *T*_max_	0.640, 0.747
No. of measured, independent and observed [*I* > 3σ(*I*)] reflections	48075, 3773, 2901
*R*_int_	0.036
(sin θ/λ)_max_ (Å^−1^)	0.772

Refinement	
*R*[*F* ^2^ > 2σ(*F* ^2^)], *wR*(*F* ^2^), *S*	0.039, 0.052, 2.73
No. of reflections	3773
No. of parameters	146
Δρ_max_, Δρ_min_ (e Å^−3^)	3.34, −2.22

Computer programs: *APEX3* and *SAINT* (Bruker, 2015 ▸), *SUPERFLIP* (Palatinus & Chapuis, 2007 ▸), *JANA2006* (Petříčcek *et al.*, 2014 ▸, *DIAMOND* (Brandenburg, 2006 ▸) and *publCIF* (Westrip, 2010 ▸).

## supplementary crystallographic information

### Crystal data

**Table:**

Na_4_Ni_7_(AsO_4_)_6_	*F*(000) = 2520
*M**_r_* = 1336.3	*D*_x_ = 4.505 Mg m^−^^3^
Monoclinic, *C*2/*m*	Mo *K*α radiation, λ = 0.71073 Å
Hall symbol: -C 2y	Cell parameters from 38754 reflections
*a* = 14.5383 (11) Å	θ = 2.1–33.3°
*b* = 14.5047 (11) Å	µ = 16.76 mm^−^^1^
*c* = 10.6120 (8) Å	*T* = 293 K
β = 118.299 (2)°	Irregular, brown
*V* = 1970.3 (3) Å^3^	0.07 × 0.06 × 0.04 mm
*Z* = 4	

### Data collection

**Table:**

Bruker D8 Venture diffractometer	2901 reflections with *I* > 3σ(*I*)
Radiation source: X-ray tube	*R*_int_ = 0.036
phi scan	θ_max_ = 33.3°, θ_min_ = 2.1°
Absorption correction: multi-scan (*SADABS*; Bruker, 2015)	*h* = −21→20
*T*_min_ = 0.640, *T*_max_ = 0.747	*k* = −22→21
48075 measured reflections	*l* = −16→16
3773 independent reflections	

### Refinement

**Table:**

Refinement on *F*	0 restraints
*R*[*F*^2^ > 2σ(*F*^2^)] = 0.039	0 constraints
*wR*(*F*^2^) = 0.052	Weighting scheme based on measured s.u.'s *w* = 1/(σ^2^(*F*) + 0.0001*F*^2^)
*S* = 2.73	(Δ/σ)_max_ = 0.015
3773 reflections	Δρ_max_ = 3.34 e Å^−^^3^
146 parameters	Δρ_min_ = −2.22 e Å^−^^3^

### Fractional atomic coordinates and isotropic or equivalent isotropic displacement parameters (Å^2^)

**Table:**

	*x*	*y*	*z*	*U*_iso_*/*U*_eq_	Occ. (<1)
Ni1	0.5	0	0	0.0068 (4)	
Ni2a	0.5	0.1306 (6)	0.5	0.0078 (8)	0.80 (4)
Ni2b	0.5	0.166 (5)	0.5	0.027 (7)	0.20 (4)
Ni3	0.91312 (4)	0.18257 (3)	0.23065 (6)	0.00542 (19)	
Ni4	0.59336 (4)	0.18693 (3)	0.26615 (6)	0.00530 (19)	
Ni5	0.5	0.32688 (6)	0	0.0112 (3)	
Ni6	0	0	0.5	0.0061 (4)	
As1	0.85957 (3)	0.18473 (3)	0.45674 (4)	0.00503 (16)	
As2	0.65106 (3)	0.18354 (3)	0.05278 (4)	0.00470 (15)	
As3	0.47038 (5)	0	0.28662 (6)	0.0062 (2)	
As4	0.45990 (5)	0.5	−0.20991 (6)	0.0056 (2)	
Na1	0.2934 (2)	0.5	−0.0189 (3)	0.0304 (13)	
Na2	0.25173 (16)	0.11586 (14)	0.3367 (2)	0.0305 (9)	
Na3	0.7399 (2)	0	0.3103 (4)	0.0435 (15)	
O1	0.6079 (2)	0.09855 (19)	0.1237 (3)	0.0067 (5)*	
O2	0.8783 (2)	0.2776 (2)	0.3717 (3)	0.0125 (6)*	
O3	0.4137 (3)	0	0.1101 (4)	0.0104 (8)*	
O4	0.4391 (2)	0.21767 (19)	0.1132 (3)	0.0090 (6)*	
O5	0.7665 (2)	0.1552 (2)	0.0682 (3)	0.0097 (6)*	
O6	0.5380 (2)	0.4081 (2)	−0.1240 (3)	0.0096 (6)*	
O8	0.3535 (3)	0.5	−0.1899 (5)	0.0140 (9)*	
O9	0.3901 (4)	0	0.3581 (5)	0.0232 (11)*	
O10	0.9333 (2)	0.20454 (19)	0.6329 (3)	0.0089 (6)*	
O11	0.5468 (2)	0.0939 (2)	0.3604 (3)	0.0115 (6)*	
O12	0.7395 (2)	0.1588 (2)	0.4274 (3)	0.0096 (6)*	
O13	0.6448 (2)	0.27433 (19)	0.1522 (3)	0.0092 (6)*	
O14	0.4220 (3)	0.5	−0.3841 (4)	0.0108 (9)*	
O15	0.8965 (2)	0.10027 (18)	0.3788 (3)	0.0062 (5)*	

### Atomic displacement parameters (Å^2^)

**Table:**

	*U*^11^	*U*^22^	*U*^33^	*U*^12^	*U*^13^	*U*^23^
Ni1	0.0070 (5)	0.0067 (5)	0.0068 (5)	0	0.0032 (4)	0
Ni2a	0.0102 (7)	0.006 (2)	0.0096 (7)	0	0.0068 (5)	0
Ni2b	0.021 (3)	0.04 (2)	0.019 (3)	0	0.010 (2)	0
Ni3	0.0047 (2)	0.0062 (3)	0.0048 (3)	−0.00029 (18)	0.0018 (2)	0.00088 (18)
Ni4	0.0050 (2)	0.0057 (3)	0.0048 (3)	0.00053 (18)	0.0020 (2)	−0.00048 (18)
Ni5	0.0097 (4)	0.0149 (4)	0.0084 (4)	0	0.0039 (3)	0
Ni6	0.0070 (5)	0.0048 (5)	0.0066 (5)	0	0.0033 (4)	0
As1	0.0049 (2)	0.0058 (2)	0.0047 (2)	0.00044 (14)	0.00248 (16)	0.00007 (14)
As2	0.00360 (19)	0.0057 (2)	0.0046 (2)	0.00079 (14)	0.00184 (16)	0.00099 (14)
As3	0.0082 (3)	0.0051 (3)	0.0054 (3)	0	0.0032 (2)	0
As4	0.0061 (3)	0.0044 (3)	0.0058 (3)	0	0.0024 (2)	0
Na1	0.0270 (17)	0.0353 (17)	0.0297 (17)	0	0.0141 (14)	0
Na2	0.0251 (11)	0.0240 (11)	0.0399 (14)	−0.0090 (9)	0.0134 (10)	−0.0080 (10)
Na3	0.0236 (17)	0.0179 (15)	0.061 (2)	0	−0.0026 (16)	0

### Geometric parameters (Å, º)

**Table:**

Ni1—O1	2.068 (2)	Ni6—O15^iv^	2.049 (2)
Ni1—O1^i^	2.068 (2)	Ni6—O15^xiii^	2.049 (2)
Ni1—O1^ii^	2.068 (2)	Ni6—O15^xiv^	2.049 (2)
Ni1—O1^iii^	2.068 (2)	As1—Na2^vii^	3.249 (2)
Ni1—O3	2.081 (6)	As1—Na3	3.1713 (16)
Ni1—O3^i^	2.081 (6)	As1—O2	1.714 (3)
Ni2a—Ni2b	0.51 (7)	As1—O10	1.681 (3)
Ni2a—Na2	3.185 (2)	As1—O12	1.664 (3)
Ni2a—Na2^iv^	3.185 (2)	As1—O15	1.702 (3)
Ni2a—O11	1.974 (4)	As2—O1	1.711 (3)
Ni2a—O11^iv^	1.974 (4)	As2—O4^i^	1.696 (3)
Ni2b—Na2	3.260 (17)	As2—O5	1.659 (3)
Ni2b—Na2^iv^	3.260 (17)	As2—O13	1.716 (3)
Ni2b—O2^v^	1.83 (3)	As3—O3	1.650 (4)
Ni2b—O2^vi^	1.83 (3)	As3—O9	1.667 (7)
Ni3—O4^vii^	2.058 (3)	As3—O11	1.697 (3)
Ni3—O5	2.047 (3)	As3—O11^iii^	1.697 (3)
Ni3—O6^viii^	2.069 (3)	As4—Na1^i^	3.240 (3)
Ni3—O10^ix^	2.029 (3)	As4—Na2^xv^	3.190 (2)
Ni3—O15	2.077 (3)	As4—Na2^xvi^	3.190 (2)
Ni4—O1	2.068 (3)	As4—O6	1.707 (3)
Ni4—O4	2.101 (3)	As4—O6^xvii^	1.707 (3)
Ni4—O10^v^	2.042 (3)	As4—O8	1.658 (6)
Ni4—O11	1.980 (4)	As4—O14	1.660 (5)
Ni4—O12	2.041 (3)	Na1—Na2^xv^	3.540 (4)
Ni5—O6	2.029 (3)	Na1—Na2^xvi^	3.540 (4)
Ni5—O6^i^	2.029 (3)	Na1—Na3^xviii^	3.923 (6)
Ni5—O13	2.097 (3)	Na1—O8	2.358 (7)
Ni5—O13^i^	2.097 (3)	Na2—Na2^iii^	3.361 (3)
Ni6—O14^x^	2.030 (6)	Na2—O2^vi^	2.294 (4)
Ni6—O14^xi^	2.030 (6)	Na2—O8^xi^	2.309 (3)
Ni6—O15^xii^	2.049 (2)	Na2—O13^vi^	2.429 (3)
			
O1—Ni1—O1^i^	92.53 (10)	Na1^i^—As4—Na2^xvi^	145.04 (5)
O1—Ni1—O1^ii^	180.0 (5)	Na1^i^—As4—O6	51.71 (9)
O1—Ni1—O1^iii^	87.47 (10)	Na1^i^—As4—O6^xvii^	51.71 (9)
O1—Ni1—O3	96.97 (12)	Na1^i^—As4—O8	132.24 (16)
O1—Ni1—O3^i^	83.03 (12)	Na1^i^—As4—O14	119.95 (18)
O1^i^—Ni1—O1^ii^	87.47 (10)	Na2^xv^—As4—Na2^xvi^	63.58 (5)
O1^i^—Ni1—O1^iii^	180.0 (5)	Na2^xv^—As4—O6	154.25 (13)
O1^i^—Ni1—O3	83.03 (12)	Na2^xv^—As4—O6^xvii^	94.48 (10)
O1^i^—Ni1—O3^i^	96.97 (12)	Na2^xv^—As4—O8	44.13 (10)
O1^ii^—Ni1—O1^iii^	92.53 (10)	Na2^xv^—As4—O14	77.65 (15)
O1^ii^—Ni1—O3	83.03 (12)	Na2^xvi^—As4—O6	94.48 (10)
O1^ii^—Ni1—O3^i^	96.97 (12)	Na2^xvi^—As4—O6^xvii^	154.25 (13)
O1^iii^—Ni1—O3	96.97 (12)	Na2^xvi^—As4—O8	44.13 (10)
O1^iii^—Ni1—O3^i^	83.03 (12)	Na2^xvi^—As4—O14	77.65 (15)
O3—Ni1—O3^i^	180.0 (5)	O6—As4—O6^xvii^	102.64 (12)
Ni2b—Ni2a—Na2	93.85 (16)	O6—As4—O8	110.86 (15)
Ni2b—Ni2a—Na2^iv^	93.85 (16)	O6—As4—O14	112.34 (14)
Ni2b—Ni2a—O11	105.6 (3)	O6^xvii^—As4—O8	110.86 (15)
Ni2b—Ni2a—O11^iv^	105.6 (3)	O6^xvii^—As4—O14	112.34 (14)
Na2—Ni2a—Na2^iv^	172.3 (3)	O8—As4—O14	107.8 (2)
Na2—Ni2a—O11	106.17 (12)	As4^i^—Na1—Na2^xv^	110.70 (11)
Na2—Ni2a—O11^iv^	71.66 (9)	As4^i^—Na1—Na2^xvi^	110.70 (11)
Na2^iv^—Ni2a—O11	71.66 (9)	As4^i^—Na1—Na3^xviii^	87.08 (8)
Na2^iv^—Ni2a—O11^iv^	106.17 (12)	As4^i^—Na1—O8	83.94 (14)
O11—Ni2a—O11^iv^	148.7 (5)	Na2^xv^—Na1—Na2^xvi^	56.69 (7)
Ni2a—Ni2b—Na2	77.1 (13)	Na2^xv^—Na1—Na3^xviii^	145.38 (8)
Ni2a—Ni2b—Na2^iv^	77.1 (13)	Na2^xv^—Na1—O8	40.16 (9)
Ni2a—Ni2b—O2^v^	116 (2)	Na2^xvi^—Na1—Na3^xviii^	145.38 (8)
Ni2a—Ni2b—O2^vi^	116 (2)	Na2^xvi^—Na1—O8	40.16 (9)
Na2—Ni2b—Na2^iv^	154 (3)	Na3^xviii^—Na1—O8	171.02 (14)
Na2—Ni2b—O2^v^	158 (2)	Ni2a—Na2—Ni2b	9.1 (13)
Na2—Ni2b—O2^vi^	43.1 (7)	Ni2a—Na2—As1^vi^	61.00 (16)
Na2^iv^—Ni2b—O2^v^	43.1 (7)	Ni2a—Na2—As4^xi^	151.93 (17)
Na2^iv^—Ni2b—O2^vi^	158 (2)	Ni2a—Na2—Na1^xi^	101.59 (12)
O2^v^—Ni2b—O2^vi^	127 (4)	Ni2a—Na2—Na2^iii^	93.85 (17)
O4^vii^—Ni3—O5	92.46 (12)	Ni2a—Na2—O2^vi^	41.55 (17)
O4^vii^—Ni3—O6^viii^	84.64 (13)	Ni2a—Na2—O8^xi^	129.3 (2)
O4^vii^—Ni3—O10^ix^	82.32 (12)	Ni2a—Na2—O13^vi^	121.44 (17)
O4^vii^—Ni3—O15	169.39 (11)	Ni2b—Na2—As1^vi^	52.1 (12)
O5—Ni3—O6^viii^	84.66 (12)	Ni2b—Na2—As4^xi^	160.9 (13)
O5—Ni3—O10^ix^	170.33 (16)	Ni2b—Na2—Na1^xi^	105.7 (6)
O5—Ni3—O15	94.46 (12)	Ni2b—Na2—Na2^iii^	102.9 (13)
O6^viii^—Ni3—O10^ix^	86.74 (12)	Ni2b—Na2—O2^vi^	33.1 (12)
O6^viii^—Ni3—O15	103.99 (12)	Ni2b—Na2—O8^xi^	137.2 (11)
O10^ix^—Ni3—O15	91.91 (12)	Ni2b—Na2—O13^vi^	114.0 (11)
O1—Ni4—O4	90.49 (11)	As1^vi^—Na2—As4^xi^	145.50 (8)
O1—Ni4—O10^v^	166.43 (12)	As1^vi^—Na2—Na1^xi^	129.31 (10)
O1—Ni4—O11	97.17 (13)	As1^vi^—Na2—Na2^iii^	152.92 (7)
O1—Ni4—O12	93.57 (12)	As1^vi^—Na2—O2^vi^	30.22 (10)
O4—Ni4—O10^v^	80.96 (11)	As1^vi^—Na2—O8^xi^	162.71 (11)
O4—Ni4—O11	92.27 (12)	As1^vi^—Na2—O13^vi^	74.79 (8)
O4—Ni4—O12	175.26 (15)	As4^xi^—Na2—Na1^xi^	69.02 (7)
O10^v^—Ni4—O11	93.70 (14)	As4^xi^—Na2—Na2^iii^	58.21 (5)
O10^v^—Ni4—O12	95.48 (12)	As4^xi^—Na2—O2^vi^	164.23 (11)
O11—Ni4—O12	84.81 (12)	As4^xi^—Na2—O8^xi^	30.00 (15)
O6—Ni5—O6^i^	108.95 (13)	As4^xi^—Na2—O13^vi^	83.39 (9)
O6—Ni5—O13	103.20 (12)	Na1^xi^—Na2—Na2^iii^	61.65 (6)
O6—Ni5—O13^i^	101.19 (12)	Na1^xi^—Na2—O2^vi^	104.27 (14)
O6^i^—Ni5—O13	101.19 (12)	Na1^xi^—Na2—O8^xi^	41.18 (16)
O6^i^—Ni5—O13^i^	103.20 (12)	Na1^xi^—Na2—O13^vi^	77.57 (10)
O13—Ni5—O13^i^	137.37 (11)	Na2^iii^—Na2—O2^vi^	132.33 (13)
O14^x^—Ni6—O14^xi^	180.0 (5)	Na2^iii^—Na2—O8^xi^	43.30 (8)
O14^x^—Ni6—O15^xii^	85.74 (12)	Na2^iii^—Na2—O13^vi^	130.98 (10)
O14^x^—Ni6—O15^iv^	94.26 (12)	O2^vi^—Na2—O8^xi^	145.4 (2)
O14^x^—Ni6—O15^xiii^	94.26 (12)	O2^vi^—Na2—O13^vi^	81.18 (12)
O14^x^—Ni6—O15^xiv^	85.74 (12)	O8^xi^—Na2—O13^vi^	88.13 (11)
O14^xi^—Ni6—O15^xii^	94.26 (12)	As1—Na3—As1^iii^	115.32 (9)
O14^xi^—Ni6—O15^iv^	85.74 (12)	As1—Na3—Na1^xix^	98.15 (10)
O14^xi^—Ni6—O15^xiii^	85.74 (12)	As1^iii^—Na3—Na1^xix^	98.15 (10)
O14^xi^—Ni6—O15^xiv^	94.26 (12)	Ni1—O1—Ni4	125.67 (17)
O15^xii^—Ni6—O15^iv^	89.57 (10)	Ni1—O1—As2	123.00 (15)
O15^xii^—Ni6—O15^xiii^	180.0 (5)	Ni4—O1—As2	93.49 (12)
O15^xii^—Ni6—O15^xiv^	90.43 (10)	Ni2b^v^—O2—As1	107.3 (16)
O15^iv^—Ni6—O15^xiii^	90.43 (10)	Ni2b^v^—O2—Na2^vii^	103.8 (19)
O15^iv^—Ni6—O15^xiv^	180.0 (5)	As1—O2—Na2^vii^	107.4 (2)
O15^xiii^—Ni6—O15^xiv^	89.57 (10)	Ni1—O3—As3	121.8 (2)
Na2^vii^—As1—Na3	120.62 (5)	Ni3^vi^—O4—Ni4	96.68 (11)
Na2^vii^—As1—O2	42.36 (13)	Ni3^vi^—O4—As2^i^	124.13 (16)
Na2^vii^—As1—O10	102.64 (10)	Ni4—O4—As2^i^	139.19 (19)
Na2^vii^—As1—O12	81.93 (11)	Ni3—O5—As2	129.51 (19)
Na2^vii^—As1—O15	130.93 (11)	Ni3^viii^—O6—Ni5	104.78 (13)
Na3—As1—O2	126.42 (12)	Ni3^viii^—O6—As4	121.23 (17)
Na3—As1—O10	127.00 (12)	Ni5—O6—As4	118.91 (19)
Na3—As1—O12	55.54 (13)	As4—O8—Na1	143.8 (2)
Na3—As1—O15	51.68 (13)	As4—O8—Na2^xv^	105.9 (2)
O2—As1—O10	105.90 (14)	As4—O8—Na2^xvi^	105.9 (2)
O2—As1—O12	119.59 (14)	Na1—O8—Na2^xv^	98.65 (19)
O2—As1—O15	98.30 (17)	Na1—O8—Na2^xvi^	98.65 (19)
O10—As1—O12	107.84 (17)	Na2^xv^—O8—Na2^xvi^	93.40 (14)
O10—As1—O15	118.79 (13)	Ni3^ix^—O10—Ni4^v^	99.49 (11)
O12—As1—O15	106.93 (14)	Ni3^ix^—O10—As1	132.3 (2)
O1—As2—O4^i^	113.78 (13)	Ni4^v^—O10—As1	122.37 (14)
O1—As2—O5	110.07 (15)	Ni2a—O11—Ni4	121.2 (3)
O1—As2—O13	98.34 (16)	Ni2a—O11—As3	100.3 (3)
O4^i^—As2—O5	114.98 (17)	Ni4—O11—As3	128.29 (18)
O4^i^—As2—O13	100.05 (14)	Ni4—O12—As1	134.0 (2)
O5—As2—O13	118.36 (14)	Ni5—O13—As2	97.57 (12)
O3—As3—O9	115.8 (2)	Ni5—O13—Na2^vii^	114.05 (13)
O3—As3—O11	112.91 (14)	As2—O13—Na2^vii^	142.98 (18)
O3—As3—O11^iii^	112.91 (14)	Ni6^xx^—O14—As4	133.6 (2)
O9—As3—O11	103.68 (16)	Ni3—O15—Ni6^xxi^	124.68 (17)
O9—As3—O11^iii^	103.68 (16)	Ni3—O15—As1	97.52 (13)
O11—As3—O11^iii^	106.83 (13)	Ni6^xxi^—O15—As1	120.85 (15)
Na1^i^—As4—Na2^xv^	145.04 (5)		

Symmetry codes: (i) −*x*+1, *y*, −*z*; (ii) −*x*+1, −*y*, −*z*; (iii) *x*, −*y*, *z*; (iv) −*x*+1, *y*, −*z*+1; (v) −*x*+3/2, −*y*+1/2, −*z*+1; (vi) *x*−1/2, −*y*+1/2, *z*; (vii) *x*+1/2, −*y*+1/2, *z*; (viii) −*x*+3/2, −*y*+1/2, −*z*; (ix) −*x*+2, *y*, −*z*+1; (x) *x*−1/2, *y*−1/2, *z*+1; (xi) −*x*+1/2, *y*−1/2, −*z*; (xii) *x*−1, *y*, *z*; (xiii) −*x*+1, −*y*, −*z*+1; (xiv) *x*−1, −*y*, *z*; (xv) −*x*+1/2, *y*+1/2, −*z*; (xvi) −*x*+1/2, −*y*+1/2, −*z*; (xvii) *x*, −*y*+1, *z*; (xviii) *x*−1/2, *y*+1/2, *z*; (xix) *x*+1/2, *y*−1/2, *z*; (xx) *x*+1/2, *y*+1/2, *z*−1; (xxi) *x*+1, *y*, *z*.
